# Effects of Partially Defatted *Hermetia illucens* Meal in Rainbow Trout Diet on Hepatic Methionine Metabolism

**DOI:** 10.3390/ani10061059

**Published:** 2020-06-19

**Authors:** Genciana Terova, Chiara Ceccotti, Chiara Ascione, Laura Gasco, Simona Rimoldi

**Affiliations:** 1 Department of Biotechnology and Life Sciences, University of Insubria, Via J.H. Dunant, 3, 21100 Varese, Italy; chiara.ceccotti@uninsubria.it (C.C.); chiaraasc@hotmail.it (C.A.); simona.rimoldi@uninsubria.it (S.R.); 2 Department of Agricultural, Forestry, and Food Sciences, University of Turin, Largo P. Braccini 2, Grugliasco, 10095 Turin, Italy; laura.gasco@unito.it

**Keywords:** aquaculture, insect meal, rainbow trout, methionine, *CBS*, *BHMT*, *SAHH*, SAM, SAH, liver

## Abstract

**Simple Summary:**

For sustainable aquaculture development, fish meal from the sea in aquafeed should be replaced with other sustainable materials such as insect larvae. The authors fed black soldier fly maggot meal to rainbow trout and examined the expression of three genes and two metabolites involved in turn-over of methionine that is an essential amino acid in fish. According to the increase in the maggot content in the aquafeed, gene expression was modulated to maintain an optimal level of methionine metabolites. Dietary replacement of up to 50% of fish meal with the maggot meal was acceptable, implying future development of a new aquafeed for sustainable aquaculture.

**Abstract:**

This study investigated, for the first time, the effects of replacement of fishmeal (FM) with insect meal from *Hermetia illucens* (HI) on the transcript levels of three genes involved in methionine (Met) metabolism in rainbow trout (*Oncorhynchus mykiss*) liver. Two target genes—betaine-homocysteine S-methyltransferase (*BHMT*) and S-adenosylhomocysteine hydrolase (*SAHH*)—are involved in Met resynthesis and the third one—cystathionine β synthase (*CBS*)—is involved in net Met loss (taurine synthesis). We also investigated the levels of two Met metabolites involved in the maintenance of methyl groups and homocysteine homeostasis in the hepatic tissue: S-adenosylmethionine (SAM) and S-adenosylhomocysteine (SAH). Three diets were formulated, an FM-based diet (HI0) and two diets in which 25% (HI25) and 50% (HI50) of FM was replaced with HI larvae meal. A 78-day feeding trial involved 360 rainbow trout with 178.9 ± 9.81 g initial average weight. Dietary replacement of up to 50% of FM with HI larvae meal, without any Met supplementation, did not negatively affect rainbow trout growth parameters and hepatic Met metabolism. In particular, Met availability from the insect-based diets directly modulated the transcript levels of two out of three target genes (*CBS*, *SAHH*) to maintain an optimal level of one-carbon metabolic substrates, i.e., the SAM:SAH ratio in the hepatic tissue.

## 1. Introduction

Aquaculture has great potential to satisfy the seafood demand from an increasing global population, which is expected to reach 9.7 billion people by 2050 [1]. However, to attain the projected growth of aquaculture, a substantial increase in the production of fish feeds will be required.

In this regard, interest in insect meal from flies, mealworms, and crickets to replace conventional protein sources in aquafeeds is continuously growing. Insects can represent a new world of sustainable and protein-rich feed and food ingredients [2]. However, although in recent years insects were often cited as “new” and “innovative”, they are not new ingredients. Rather, these ingredients have been used for millennia and have been an integral part of traditional food culture in many world regions. Nevertheless, modern Europe never became entomophagous [3].

Recently, insects have emerged as a new sector in the food and feed industry as new protein sources to replace a reasonable portion of the animal proteins consumed, mainly because of the many environmental benefits. There are several major environmental advantages of insect farming over livestock production, including less water and arable land being required, lower greenhouse gas emissions, and higher feed conversion efficiency. Furthermore, insects can feed on substrates of low value, such as bio-waste and organic side streams (which would otherwise end up in dumpsites, causing environmental pollution) and can transform them into a high-value food and feed resource, promoting the concept of a circular economy and zero waste. Therefore, insects represent a serious alternative to other animal-based protein sources for conventional production, either for direct human consumption or indirectly as feedstock [3,4].

Recently, an European Union (EU) commission regulation (2017/893-24/05/2017) authorized seven insects (two flies, two mealworms, and three cricket species) for fish feed. Of these, black soldier fly, *Hermetia illucens* (HI), which belongs to the Diptera order, is one of the most promising species. Replacing up to 50% of fishmeal (FM) with HI meal has proven to not negatively affect growth performance and feed digestibility of different fish species including Atlantic salmon (*Salmo salar*), rainbow trout (*Oncorhynchus mykiss*), European sea bass (*Dicentrarchus labrax*), Jian carp (*Cyprinus carpio* var. Jian), and yellow catfish (*Pelteobagrus fulvidraco*) [5,6,7,8,9,10]. Moreover, HI meal has shown a positive effect on fish gut microbiota, increasing the amount of beneficial lactic acid- and butyrate-producing bacteria [11,12].

In terms of protein quality, HI larvae meal has a well-balanced amino acid profile that is closely comparable to that of soybean meal (SBM) and only slightly less optimal than wild-caught FM. In particular, levels of lysine, histidine, threonine, and tyrosine (in % of protein) are higher in HI meal than in SBM. If we consider the interrelation between sulfur-containing amino acids such as methionine (Met) and cysteine (Cys), however, levels are lower in HI meal than in SBM and FM [3,13].

Fish cannot synthesize Met de novo at a rate commensurate with their demand and thus this amino acid must be supplied in their diet. Met is often the first limiting amino acid in many fish diets, especially in those containing high levels of plant proteins derived from soya and pea, but this can also be the case in diets with high levels of FM replacement with HI meal. A relative deficiency of Met requires that this amino acid be supplemented with, for example, crystalline Met to achieve a satisfactory profile that meets fish dietary requirements [14,15,16,17,18]. The dietary Met requirements have been estimated for commonly cultivated species of fish, ranging from 1.8 to 4.0% of dietary protein [19].

Met and its intermediate metabolites S-adenosylmethionine (SAM) and S-adenosylhomocysteine (SAH) play a pivotal role in several biological processes. By generating SAM that serves as the main methyl group donor for the majority of cellular methylation reactions, Met participates in the methylation of DNA, ultimately influencing gene expression [20]. In addition to its role in protein synthesis, Met is involved in fatty acid oxidation, phospholipid status, creatine synthesis, bile acid production, and polyamine (i.e., spermidine and spermine) biosynthesis [21]. Met is also precursor of taurine (an osmolyte, i.e., a low-molecular-weight organic compound that influences the properties of biological fluids) and Cys, one of the three amino acids constituting glutathione (γ-l-glutamyl-l-cysteinyl-glycine), which is the most important low-molecular-weight antioxidant synthesized in cells [20,21].

Nearly half of the Met metabolism (48%) and up to 85% of all methylation reactions take place in the liver [22]. Therefore, dietary and hepatic Met levels are tightly associated [23].

In liver, two intersecting pathways, remethylation and transsulfuration, compete for homocysteine (Hcy), a sulfur-containing metabolite that is formed from the demethylation of Met. Remethylation of Hcy functions to conserve Met. In contrast, transsulfuration serves to irreversibly catabolize Hcy while synthesizing Cys [24].

Following the transfer of a methyl group to an acceptor molecule, SAM is converted to SAH [22,24,25,26], which is further converted into Hcy and adenosine. The SAM/SAH ratio (often used as an indicator for intracellular methylation capacity) is affected by the activity of S-adenosylhomocysteine hydrolase (SAHH), which hydrolyzes SAH to Hcy and adenosine. The SAHH reaction is reversible, but under normal physiologic conditions, it proceeds in the hydrolytic direction due to the efficient removal of Hcy [26].

Two main enzymes involved in removing Hcy are cystathionine β synthase (CBS), which catalyzes the condensation of Hcy and serine to cystathionine, and betaine-homocysteine S-methyltransferase (BHMT), which transfers the methyl-group of betaine to re-methylate Hcy and regenerate Met.

BHMT and CBS have different affinities for their substrate (Hcy): BHMT has a high affinity (low Michaelis constant, KM, values) and can utilize Hcy at a relatively low concentration [27], whereas CBS has low affinity in the catabolic pathway. Thus, dysfunction of CBS causes higher levels of Hcy in the blood than does BHMT. Accordingly, the dietary uptake of either deficient or adequate Met levels could provoke alterations in the SAM/SAH ratio in the animal, resulting in a deregulation of cellular homeostasis and health issues [21].

The results of previous research have illustrated a tight connection between dietary Met intake and one-carbon metabolic substrates, i.e., SAM and SAH, in liver of different animals [23]. Generally, an increase in hepatic SAM is expected when dietary Met intake is increased, but in some mammals (rodents), hepatic SAM levels were maintained, whereas SAH gradually increased with the increase in dietary Met, leading to a reduction in the SAM/SAH ratio.

Accordingly, the aim of the present experiment was to investigate in rainbow trout (*Oncorhynchus mykiss*) the effect of two replacement levels (25% and 50%) of FM with insect meal from *H. illucens* larvae, without Met supplementation, on the hepatic SAM/SAH ratio and on the mRNA levels of genes *SAHH*, *BHMT*, and *CBS* involved in Met metabolism.

The present study deals with a smaller component of a larger study, and the results on growth performance, somatic indexes, and intestinal morphology of rainbow trout fed with *H. illucens* larvae meal, have been presented by Renna et al. [8]. The point of difference and the novelty of the current work are the gene expression component and the study of the intermediates metabolites of the Met pathway, i.e., SAM and SAH, in fish fed diets with insect meal. Only details vital to this manuscript are repeated in detail in the text whilst referencing Renna et al. [8] for other data/information.

## 2. Materials and Methods

The feeding trial was conducted at the Experimental Facility of the Department of Agricultural, Forest, and Food Sciences (DISAFA) of the University of Torino (Italy). The experimental protocol was designed according to the guidelines of the European Union Council (2010/63/EU) (European directive 86,609/EEC, put into law in Italy with D.L. 116/92) for the use and care of experimental animals. For details on the experimental protocol and chemical analysis of the diets, please refer to Renna et al. [8].

### 2.1. Gene Expression Analysis

#### 2.1.1. Fish Sampling

Rainbow trout (*O. mykiss*) of about 180 g were fed three experimental diets, named HI0, HI25, and HI50. Diets were formulated to contain increasing levels of partially defatted HI larvae meal (0%, 25%, and 50%) to replace the same percentage of FM. A diet with 0% inclusion of HI meal (HI0) that was an FM-based diet, served as the control. HI meal was obtain by HI larvae reared on vegetable by-product substrate. HI larvae meal was partially defatted using a mechanical process (high pressure) without solvents [8].

At the end of the feeding trial, eight fish/dietary group (two fish/tank) were sampled for gene expression and HPLC analysis. For this, fish were caught and immediately euthanized with an overdose of anesthetic (200 mg/L of MS222). Dead fish were removed with the aid of a net from the anesthetic solution and placed on a white towel. Then, the liver was aseptically removed from each fish. Liver tissues destined for gene expression analysis were stored in RNALater at −20 °C, whereas liver sections destined for HPLC analysis were snap frozen and stored at −80 °C.

#### 2.1.2. Total RNA Extraction and cDNA Synthesis

Total RNA was extracted from each sample of rainbow trout liver using an automatic system (Maxwell^®^ 16 Instrument, Promega, Milan, Italy) and a total RNA purification kit (Maxwell^®^ 16 Tissue LEV). Briefly, 125 mg of each tissue was homogenized using GentleMACS Dissociator (Miltenyi Biotec, Milan, Italy) with ice-cold Lysis Solution containing 1-thioglycerol until no visible tissue fragments remained. The same volume of RNA Dilution Buffer was added to the lysate, and the whole volume was mixed by vortex and then transferred into Maxwell^®^ 16 LEV Cartridges for RNA isolation.

The quantity of the total RNA extracted was calculated by measuring the absorbance at 260 nm using a NanoDrop™ 2000c spectrophotometer (Thermo Scientific, Milan, Italy), whereas RNA integrity was verified by agarose gel electrophoresis. The purity of RNA was assessed at 260/280 nm and averaged 2.10 ± 0.01.

After extraction, 3 µg of total RNA was reverse transcribed into cDNA in a mix of a volume of 20 μL containing oligo dT16 primers, as described in the SuperScript III reverse transcriptase kit (Invitrogen, Milan, Italy).

#### 2.1.3. Generation of in Vitro-Transcribed mRNAs for Standard Curves

Two sets of forward and reverse primers were designed based on Atlantic salmon *BHMT* (betaine-homocysteine methyltransferase) and *SAHH* (S-adenosylhomocysteine hydrolase) cDNA sequences available in the NCBI database (GenBank accession number: BT043706 and NM_001140375, respectively). Another set of primers was designed for cystathionine-beta-synthase (*CBS*) based on the rainbow trout cDNA sequence available in the NCBI database (GenBank acc. nr.: NM_001124686). To generate the in vitro-transcribed mRNAs for *BHMT*, *SAHH*, and *CBS*, liver cDNA was amplified via PCR, using for each gene a forward primer engineered to contain a T7 phage polymerase promoter gene sequence at its 5’ end and a reverse primer (Table 1). PCR products were then run on a 2% agarose gel stained with ethidium bromide. PCR products were subsequently cloned using the pGEM^®^-T cloning vector system (Promega, Milan, Italy) and then sequenced in the SP6 and M13 directions.

In vitro transcription was performed using T7 RNA polymerase and other reagents supplied in the RiboProbe In Vitro Transcription System kit (Promega, Milan, Italy) following the manufacturer’s protocol.

The synthetic mRNAs obtained by in vitro transcription were used as quantitative standards in the real-time PCR analysis of the biological samples. For this, defined amounts of mRNAs at 10-fold dilutions were subjected to real-time PCR using iTaq™ Universal Probes One-Step kit (Bio-Rad, Milan, Italy) and the following RT-PCR conditions: 10 min at 50 °C (reverse transcription reaction), 3 min at 95 °C, followed by 40 cycles consisting of 15 s at 95 °C and 1 min at 60 °C. The Ct values obtained by amplification were used to create standard curves for target gene quantification.

#### 2.1.4. Transcript Quantification by One-Step TaqMan^®^ real-time RT-PCR

One hundred nanograms of total RNA extracted from the trout livers was subjected to One-step Taqman^®^ quantitative real-time RT-PCR. Biological samples were loaded in the same 48-well plate with standard mRNAs and run under the same aforementioned real-time RT-PCR conditions.

Real-time Assays-by-DesignSM PCR primers and gene-specific fluorogenic probes were designed by Invitrogen, Milan, Italy. Primer sequences and TaqMan^®^ probes used for each target gene amplification are shown in Table 1. TaqMan^®^ PCR reactions were performed on a Bio-Rad^®^ CFX96™ System.

Data from the TaqMan^®^ PCR runs were collected with Bio-Rad^®^ Software. Ct values corresponded to the number of cycles in which the fluorescence emission monitored in real-time exceeded the threshold limit. The Ct values were used to create standard curves to serve as a basis for calculating the absolute amount of mRNAs in total RNA for each gene.

### 2.2. SAM/SAH HPLC Analysis

#### 2.2.1. Reagents

S-Adenosylmethionine (SAM) and S-Adenosylhomocysteine (SAH) standards were obtained from Sigma Aldrich (St. Louis, MO; USA).

Methanol, perchloric acid (70%), and orthophosphoric acid (85%) were purchased from Carlo Erba Reagents S.r.l (Milan, Italy). All reagents were characterized by the highest purity grade.

#### 2.2.2. Standards and Biological Sample Preparation

SAM and SAH standards were first prepared in HPLC water at 1 mM concentration and then sequentially diluted with 0.4 M perchloric acid (HClO_4_). To ensure a good calibration curve, five concentrations were prepared.

To extract biological samples, 0.2 g of fish liver (wet tissue) was mixed with 800 uL of perchloric acid (0.4 M) and homogenized for 4 min using a TissueLyser II (Qiagen, Milan, Italy) at 4 °C with stainless steel beads of 5 mm diameter. After centrifugation at 10,000 g, for 15 min at 4 °C, the supernatant was collected and then filtered into vials through a 0.22-μm syringe filter. The extracted solution was stored at −80 °C until HPLC analysis.

#### 2.2.3. HPLC System and Chromatographic Conditions

The HPLC system consisted of a PU-2089 quaternary pump connected to a degasser and a MD-2015 diode array, both obtained from Jasco-Europe S.r.l Company (Milan, Italy).

Separation was carried out in a Kinetex XB-C18 reversed-phase analytical column (250 mm x 4.6 mm, length × internal diameter) with a particle size of 5 μm (Phenomenex, Milan, Italy). A HPLC universal guard column (Phenomenex, Milan, Italy) protected the HPLC column. Solvent A and B formed the gradient elution.

Solvent A was an aqueous buffer containing 8 mM octanesulfonic acid sodium salt and 50 mM NaH2PO4, and it was adjusted to pH 3 with H3PO4. Solvent B consisted of 100% methanol. Solvent A was filtered through a 0.2-μm membrane filter.

The column was conditioned with 80% solvent A and 20% solvent B. The first step gradient consisted of 8 min at the equilibration condition, 4 min to increase solvent B to 25%, followed by a further increase of the same solvent to 30%. Subsequently, the ramp was an increase to 40% of solvent B for 4 min. The ratio 60% solvent A: 40% solvent B was maintained for 12.5 min, followed by 30 s to return to equilibration conditions (80% solvent A: 20% solvent B, and 10 min to finish the elution and return to the initial conditions.

The analysis of standards and biological samples was performed at room temperature. A volume of 20 μL solution was injected into HPLC. The flow rate was set at 0.9 mL/min, and the detector was set at a wavelength of 254 nm. Data processing was performed using ChromNAV (Jasco-Europe, city, country).

SAM and SAH were quantified by comparing their peak area to the relative peak areas of the standard calibration curves. The aforementioned method is a slightly modified version of the method applied by Wang et al. [28] to extract and quantify SAM-SAH in trout liver.

#### 2.2.4. Validation

For the validation, each biological sample and standard were run three times. A blank sample was injected daily at the beginning of analysis to check the baseline and to monitor the matrix effect due to sample injection. A blank was also run between each sample to ensure there was no memory. The equation of the calibration curves showed r^2^ = 0.998 for SAM and r^2^ = 0.997 for SAH.

### 2.3. Statistical Analyses

The growth performance and the Met metabolism data were analyzed by one-way analysis of variance (ANOVA) using STATISTICA software, StatSoft^®^ Europe (Hamburg, Germany). If there were significant differences between dietary groups, Tukey’s test was applied as post hoc analysis. The level of statistical significance was set at *p* < 0.05.

## 3. Results

### 3.1. Fish Performance

Renna et al. [8] reported fish growth, somatic indexes, and condition factor data. However, briefly, at the end of the 78-day feeding trial, fish tripled their initial weights but no differences were detected in weight gain, condition factor, and the specific growth ratio (SGR) between HI0 and the HI25 and HI50 dietary groups. The feed conversion ratio (FCR) was low (less than 1) in all experimental groups. As for the somatic indexes, non-significant differences were recorded for carcass yield, the hepatosomatic- and viscerosomatic index (HSI, VSI), and coefficient of fatness between the control and experimental trout dietary groups.

### 3.2. Gene Expression

**Figure 1:**
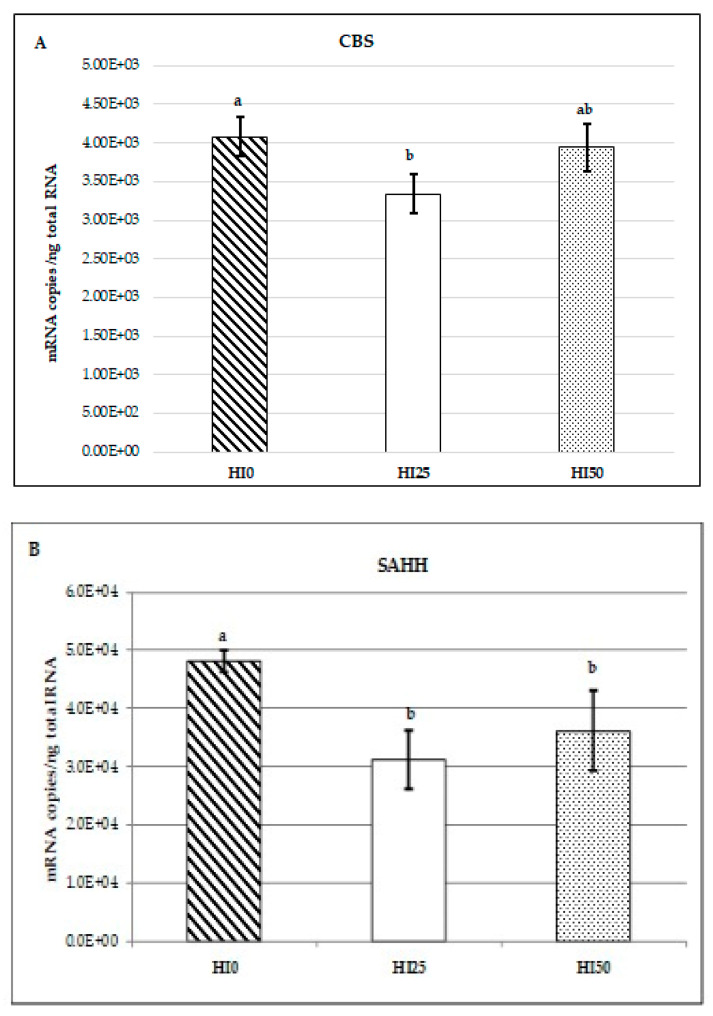

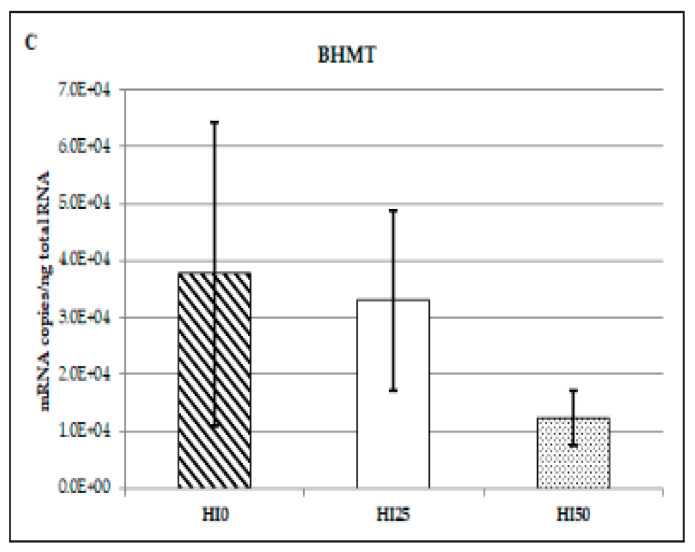
(**A**–**C**). Transcript copies of genes coding for *CBS*, *SAHH*, and *BHMT* enzymes in the liver of rainbow trout fed a control diet with no inclusion of HI meal and two other diets with 25% (HI25) and 50% (HI50) inclusion. Different letters indicate significant differences (*p* < 0.05). Data represent the mean ± s.d. (n = 8).

The *CBS* mRNA copy number was significantly lower (*p* < 0.05) in the liver of trout fed the HI25 diet than in fish fed the control (HI0) diet, whereas the *CBS* transcript levels in fish fed the HI50 diet were not different from the other two fish dietary groups (Figure 1A). The *SAHH* gene showed the same trend as that of *CBS*, with the lowest number of mRNA copies recorded in the HI25 fish group (*p* < 0.05; Figure 1B). However, unlike *CBS*, the expression of the *SAHH* gene in the HI25 group was not statistically different from that of the HI50 group. The expression levels of the *BHMT* gene were not significantly different between the fish feeding groups (*p* > 0.05; Figure 1C).

### 3.3. Hepatic SAM and SAH Concentrations

The means of hepatic SAM concentrations were significantly higher in the HI50 group (65.71 nmol/g) than in both the HI0 (59.39 nmol/g) and HI25 (61.17 nmol/g) dietary groups, with no differences between HI0 and HI25. (Table 2). The HI50 group also showed the lowest hepatic SAH concentration (17.28 nmol/g) with respect to the other two groups. A tendency for SAH concentrations to decrease with increasing levels of HI meal in the diet was recorded. Furthermore, hepatic SAH concentrations increased linearly as SAM concentrations decreased. Consequently, the highest SAM/SAH ratio was detected (*p* < 0.001) in the liver of fish fed the HI50 diet, whereas the lowest ratio was recorded in the liver of fish fed the HI0 diet.

## 4. Discussion

The fast growth of aquaculture is challenging feed producers and the research community to reduce pressure on wild capture fisheries and to find ecologically friendlier and cost-effective alternatives for aquafeeds [1]. One option being explored is rearing terrestrial invertebrates such as flies for aquaculture feed production [13]. *Hermetia illucens* (HI), also known as black soldier fly, is currently one of the most widely used insect meals for fish feed, and standard mass-rearing techniques for the industrial production of this species are already available [29]. HI larvae meal has a well-balanced aa profile, but levels of sulfur-containing amino acids, Met + Cys, are sometimes lower than those in FM, yet higher than those in SBM, which is the plant protein that is currently the most widely used to replace FM in fish feeds [3,13].

In rainbow trout, the Met + Cys requirement ranges between 2.2 and 3.0% of the dietary protein [14,15,16,17,18,30,31,32]. Adding Cys to the diet reduces the dietary Met requirements because Met can be converted to Cys [33]. Although insect meals are generally characterized by lower levels of Met + Cys than in FM (depending on insect species), the HI meal used in the present experiment met trout requirements, containing 2.1% of Met and 0.1% of Cys. Usually, FM contains Met at the level of 2.8% of dietary protein and Cys at 0.8%.

The present feeding trial using dietary HI meal did not have any negative effects on rainbow trout performance. Indeed, values of SGR, FCR, and PER (protein efficiency ratio) indexes as well as the fillet lipid content provided evidence of good nutrient bioavailability, as reported in Renna et al., [8].

With regard to the hepatosomatic index (HSI), the lack of differences between the control (1.63) and trout fed the HI25 (1.73) and HI50 (1.71) diets is a good result, as an increase in this index would be due to glycogen and/or lipid deposition in the liver, as shown in trout by Walton et al. [34]. Our results showed that Met levels were optimal in all the diets (Met varied from 2.1 to 2.7, which is not a large difference); other studies have shown that diets low in Met negatively affected liver lipid metabolism in salmon and trout, increasing the lipid stores and contributing to a higher relative liver weight [35,36]. Indeed, there are several intersections between hepatic Met metabolism and lipid metabolism, including metabolic pathways involved in lipoprotein secretion and in de novo lipogenesis [35,36].

On the other hand, a decrease in HSI would be due to a combination of reduced voluntary feed intake in fish and imbalanced amino acid profiles of the diets, as shown by Dias et al. [37]. Therefore, the lack of differences in HSI between the dietary fish groups indicates that, in addition to an optimal level of Met, no problems arose because of HI consumption by trout in the present trial.

In contrast, Lock et al. [5] found an increase in HSI (most likely due to a higher hepatic fat content) when FM was substituted by 100% HI meal in salmon diets. In our opinion, this differing result can be ascribed to the higher level of saturated fatty acid due to the much higher level of HI included in the salmon diet, but other factors could have been involved in liver fat accumulation as well.

Met is mainly metabolized in the hepatic tissue of all living animals [28]. In this pathway, Met generates SAM, which is converted to SAH after transfer of its methyl group. SAH is then hydrolyzed to form Hcy, which can be either remethylated to form Met or enter the transsulfuration pathway to form Cys. Entry of Hcy into the transsulfuration pathway is controlled by the cystathionine b-synthase (CBS) enzyme that catalyzes the β-replacement of the hydroxyl group of Ser with Hcy, forming cystathionine with the release of water [38,39].

*CBS* mRNA is abundant only in the liver and kidney, although it can be detected at much lower levels in other tissues [38]. The expression of *CBS* is regulated by dietary Met availability. In a study conducted in mouse, Prudova et al. (2006) [40] showed that levels of CBS protein decreased 10-fold under conditions of Met restriction, as a result of lower levels of SAM, which operates as an allosteric activator of CBS by binding to the C-terminal regulatory domain [39,40,41]. In contrast, we found the lowest mRNA copy number of the *CBS* gene in the liver of trout fed the HI25 diet, whereas the expression levels in trout fed the lowest Met-containing diet (HI50) were slightly higher (although not significantly) than those in HI25 and similar to the control fish fed the HI0 diet. This can be due to interspecific differences in Met metabolism between fish and mammals. Our outcome is in line with a study conducted in fish by Kwasek et al. (2014) [22], in which Atlantic salmon (*Salmo salar*) fed a low Met diet showed higher mRNA copies of the *CBS* gene than fish receiving higher Met levels. This might indicate that remethylation was downregulated in the liver of salmon in the case of Met deficiency, suggesting that dietary Met is converted directly into Cys and then into taurine to maintain the taurine levels required for biological processes [20]. However, future studies are required to address the possible contribution of other molecular mechanisms that may increase *CBS* expression or activity, such as the ratio of Cys to Met in the diet [42], or levels of certain dietary amino acids, e.g., glycine and serine that participate in the metabolism of Hcy and thereby enhance the removal of Hcy [43].

Another gene that we targeted was *BHMT*, which encodes for a key Hcy catalyzing enzyme. As is known, Hcy metabolism involves transsulfuration or remethylation. During transsulfuration, Hcy is irreversibly converted into Cys by CBS, whereas during remethylation, Hcy is methylated to Met by methionine synthase or by BHMT. Augmented transsulfuration is the means by which excessive Met is catabolized. Conversely, Met can be conserved by increasing Hcy remethylation relative to transsulfuration via Cys synthesis [25,33]. We focused on the *BHMT* gene since studies in rodents indicated that low Met diets can increase *BHMT* gene expression [44]. It has also been shown that the relative contribution of BHMT to Hcy remethylation can be affected by diet [45]. However, in our study, the expression of *BHMT* in the liver of rainbow trout was not affected by dietary treatments, suggesting that Met levels were adequate in all the diets.

Our study is the first to compare the intermediates metabolites of the Met pathway, i.e., SAM and SAH, in fish fed with diets including insect meal.

SAM (also known as AdoMet) is a sulfonium molecule synthesized from ATP and Met, whose major function is to serve as a methyl donor for SAM-dependent methyltransferases in all living organisms. Therefore SAM is involved in many biochemical processes, the one best known being methylation, which occurs in DNA, RNA, proteins, phospholipids, hormones, and neurotransmitters [46]. In humans, the estimated half-life of hepatic SAM is 2.4–5.9 min under normal dietary conditions, and a normal adult makes approximately 6–8 g of SAM per day [47].

When SAM is used for the methylation reaction, SAH is formed. SAH is a potent, nonselective feedback inhibitor of SAM-dependent methyltransferases; therefore, to mitigate its toxicity, it is degraded into adenosine and Hcy by SAH hydrolase (SAHH). The intracellular ratio of SAM to SAH is an important index of transmethylation potential. Lower levels of SAM and higher levels of SAH may result in a reduced methylation capacity, which is represented by a low SAM/SAH ratio.

In mammals, the results of studies related to hepatic SAM and SAH levels have been controversial. In rats, SAM and SAH were markedly elevated by excess dietary Met according to the study of Rowling et al. [48]. Changes in SAM and SAH (increased SAM, a much higher increase in SAH) were such that both rat groups receiving excess dietary Met showed similar, six-fold elevations in the SAM:SAH ratio compared to controls. In another study conducted in rats by Shivapurkar and Poirier [49], the administration of Met-deficient diets for up to five weeks led to decreased hepatic SAM, an increased SAH content, and, therefore, decreased SAM:SAH ratios. Linear regression analysis showed a significant, direct correlation between the observed hepatic SAM levels and the methyl content of the diet as well as an inverse correlation between hepatic SAH levels and dietary methyl contents [49].

In agreement with the study of Shivapurkar and Poirier [49], we detected significantly high hepatic SAM and SAM:SAH ratio levels in trout fed a low-Met diet (HI50) with respect to control fish fed the HI0 diet. Our results are also in line with another study conducted in fish by Espe et al. [50]. These authors found higher hepatic SAM levels in juvenile Atlantic salmon fed a Met-deficient diet as well as a high SAM/SAH ratio (SAH concentrations were not affected), suggesting that the methylation capacity of the liver was elevated.

With regard to the mRNA levels of the *SAHH* enzyme, we found much higher copies in the liver of the HI0 fish group than in the other two groups fed lower levels of Met. In the HI0 group, the increased expression of the *SAHH* gene was paralleled by the higher hepatic SAH level with respect to the HI25 and HI50 groups. This result is once again in agreement with the outcomes of Kwasek et al. [22], who showed an increase in *SAHH* gene expression in fish fed a Met-supplemented diet and, as a consequence, an accumulation of SAH due to dietary Met intake.

## 5. Conclusions

The present study showed that dietary replacement of up to 50% of FM with insect meal from *H. illucens* larvae, without any Met supplementation, did not negatively affect hepatic Met metabolism in rainbow trout (*O. mykiss*). In particular, Met availability in the insect-based diets directly modulated the transcript levels of two (*CBS*, *SAHH*) out of three target genes (*CBS*, *SAHH*, and *BHMT*) involved in the Met pathway, maintaining an optimal level of one-carbon metabolic substrates, i.e., SAM:SAH ratio in the hepatic tissue.

Based on these findings, future trials should address the HI effects on the hepatic protein levels and activity of CBS, SAHH, BHMT, and other enzymes such as methionine adenosyltransferase (MAT) to verify whether they develop in parallel with the increase/decrease in mRNA copy number.

## Figures and Tables

**Table 1:** Sequences of the primers used for molecular cloning and one-step quantitative real-time RT-PCR (reverse transcription polymerase chain reaction).

Gene	Nucleotide Sequence (5’→3’)	Purpose
*BHMT FW*	TGCAGAGTACTTTGAGCACGT	Cloning
*BHMT RV*	CCGTGACTACTGGGAGAAGC	
*SAHHB FW*	CCCTTCAAAGTTGCTGACATCA	
*SAHHB RV*	ATGTGTGGTGCATTGAGCAGA	
*CBS FW*	AAACCCTGGTGGTGGAAC	
*CBS RV*	GTGCTCTACAAACAATTCAAACAGGT	
*T7 BHMT sense*	gtaatacgactcactatagggTGAAAGAGGGAGTGGAGAGG	Standard Curve
*BHMT antisense*	CCGTGACTACTGGGAGAAGC	
*T7 SAHHB sense*	gtaatacgactcactatagggAGATGAGGGAGCTGTATGGC	
*SAHHB antisense*	ATGTGTGGTGCATTGAGCAGA	
*T7 CBS sense*	gtaatacgactcactatagggAAACCCTGGTGGTGGAAC	
*CBS antisense*	GTGCTCTACAAACAATTCAAACAGGT	
*BHMT FW*	TGCCAGGGATTCATCGATCTG	Real-time RT-PCR
*BHMT RV*	ATGACCAGGTGGGACATGCAC	Amplicon size: 75 bp; E = 91%; R2 value = 0.99
*BHMT probe*	AGAATTCCCCTTCGGTCTGGAGCCCA	
*SAHHB FW*	CCGCCGTGCTCATTGAGA	Amplicon size: 65 bp; E = 93% R2 value = 0.99
*SAHHB RV*	GTTCAATGGTCCAGCTGCAATATC	
*SAHHB probe*	CTGCCCTTGGAGCCGA	
*CBS FW*	AGACCATCAAGATCCTCAAGGAGAA	Amplicon size: 62bp; E = 94%; R2 value = 0.99
*CBS RV*	TCGTTGACGAGTCCGGC	
*CBS probe*	GGCTTTTGACCAGG	

**Table 2:** Liver concentrations of SAM and SAH and the SAM/SAH ratio measured in rainbow trout (n = 8). Different letters in the same row indicate statistically significant differences (*p* < 0.05): one-way ANOVA followed by Tukey’s test.

Hepatic Concentrations	Diets			SEM	*p*-Value
	HI0	HI25	HI50		
SAM nmol/g	59.39 ^b^	61.17 ^b^	65.71 ^a^	0.88	*p* < 0.001
SAH nmol/g	37.81 ^a^	26.64 ^b^	17.28 ^c^	2.58	*p* < 0.001
SAM/SAH nmol/g	1.57 ^c^	2.32 ^b^	3.82 ^a^	0.28	*p* < 0.001

HI, *Hermetia illucens*; SEM, standard error of the mean; p, probability; S-Adenosylmethionine (SAM); S-Adenosylhomocysteine (SAH).
